# Supplementary material for: The behavioral signature of stepwise learning strategy in male rats and its neural correlate in the basal forebrain
Source: Nat Commun. 2023 Jul 21;14:4415. doi: 10.1038/s41467-023-40145-9 (PMC10362048; doi:10.1038/s41467-023-40145-9)
Supplement: Supplementary file 1 — Supplementary Information [file 41467_2023_40145_MOESM1_ESM.pdf]

Supplementary Figures

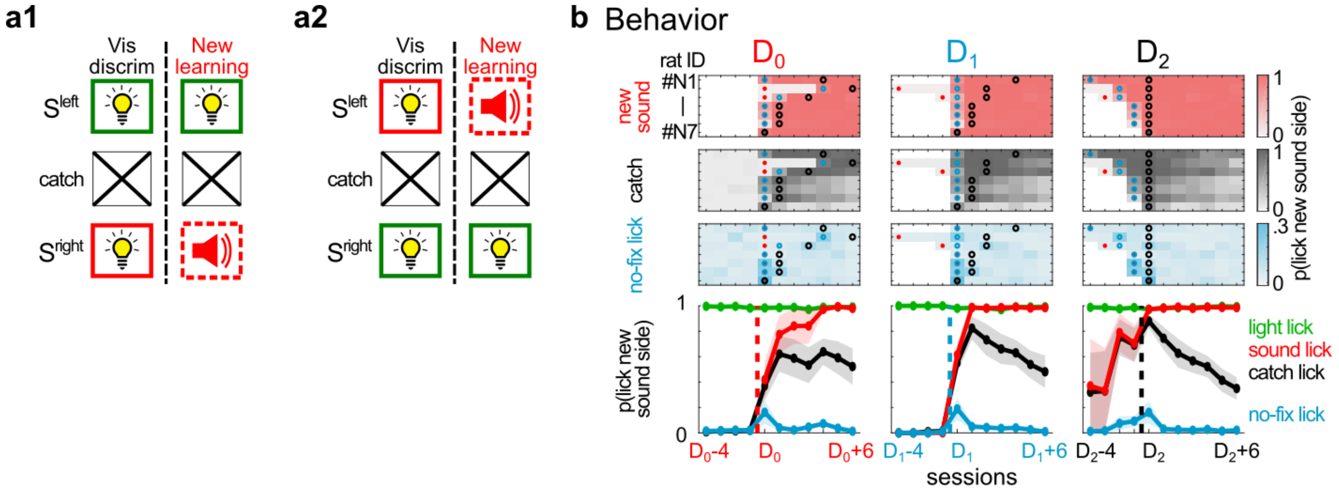

c

	Visual discrimination phase			New learning phase	
Animal ID	S-left	S-right	Group	S-left	S-right
#N1	1s light above LEFT reward port	1s light above RIGHT reward port	A2	**1s 6kHz sound, center speaker**	1s light above RIGHT reward port
#N2	1s light above LEFT reward port	1s light above RIGHT reward port	A1	1s light above LEFT reward port	**1s 6kHz sound, center speaker**
#N3	1s light above LEFT reward port	1s light above RIGHT reward port	A2	**1s 6kHz sound, center speaker**	1s light above RIGHT reward port
#N4	1s light above LEFT reward port	1s light above RIGHT reward port	A1	1s light above LEFT reward port	**1s 6kHz sound, center speaker**
#N5	1s light above LEFT reward port	1s light above RIGHT reward port	A2	**1s 6kHz sound, center speaker**	1s light above RIGHT reward port
#N6	1s light above LEFT reward port	1s light above RIGHT reward port	A2	**1s 6kHz sound, center speaker**	1s light above RIGHT reward port
#N7	1s light above LEFT reward port	1s light above RIGHT reward port	A1	1s light above LEFT reward port	**1s 6kHz sound, center speaker**

Figure S1. Consistent behavioral response patterns during new learning under variations of stimulus modalities and response directions

a, A separate cohort of 7 rats were divided into two groups, depicted in a1 and a2. Both groups initially learned a visual discrimination task, with lights above the left or right reward port serving as the sensory cue for reward in the respective port. At the new learning phase, one of the two lights was replaced by a 6 kHz (70dB) sound, delivered from a speaker placed above the center fixation port. **a1**, In this group, the S^{right} light was replaced (N=3). **a2**, in this group, the S^{left} light was replaced (N=4). All other elements of the task remained the same. Symbols were adapted from Avila I, Lin SC (2014) Motivational Salience Signal in the Basal Forebrain Is Coupled with Faster and More Precise Decision Speed. PLOS Biology 12(3): e1001811. https://doi.org/10.1371/journal.pbio.1001811. **b**, The proportion of the three types of reward-seeking behaviors toward the reward port associated with the new sound stimulus side during new learning (mean ± s.e.m., N=7 rats). Conventions as in Figure 2c. Similar to the results in Figure 2c, no-fixation licks were most consistently associated with the D_1 session, which occurred earlier than the peak of catch licks (D_2 session). Further, the proportion of sound licks and catch licks were highly similar prior to the D_2 session, and began to diverge at the D_2 session. Thus, the pattern of sequential refinements can be observed regardless of the sensory modality of the stimulus or the laterality of the new learning side. **c**, Stimulus parameters of the S^{left} and S^{right} stimuli for each animal in this cohort.

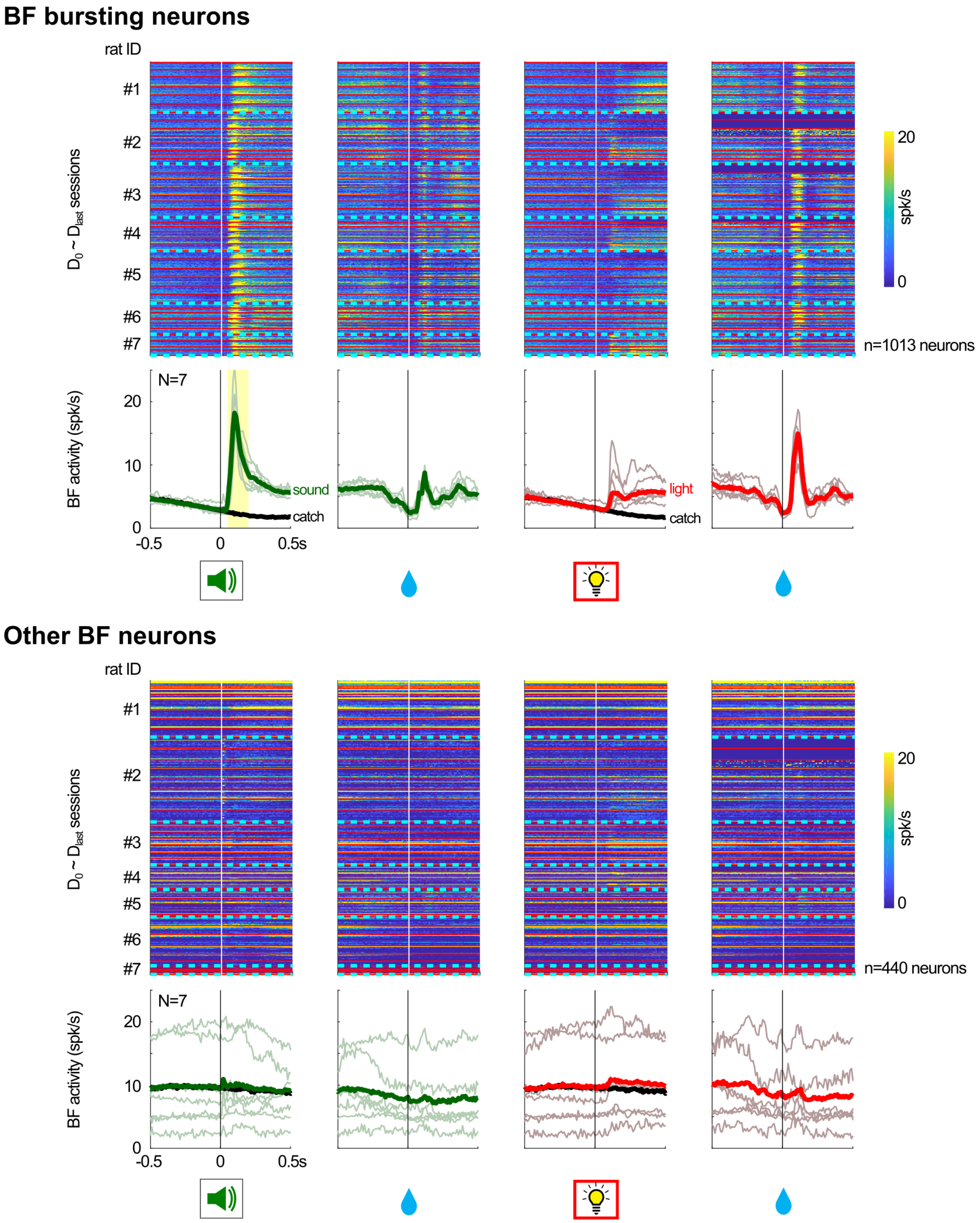

Figure S2. Identification of BF bursting neurons and their responses to behavioral events

Responses of individual BF bursting neurons (n=1013) and other BF neurons (n=440) to

behavioral events (stimulus onset and reward) in the S^{left} sound trials (two left columns) and in the S^{right} light trials (two right columns). Recordings from different sessions (N=45 sessions; separated by thin red lines) and different animals (N=7 rats; separated by cyan dotted lines) were separated by horizontal lines. Lower panels showed the average response (thick lines) pooled across individual animals (thin lines). BF activities in catch trials (thick black lines) were plotted for comparison. Responses to the stimulus onset event were calculated based on all trials in that session, regardless of subsequent behavioral responses (licking or not). On the other hand, responses to the reward were calculated based only on correct licking trials. Conventions as in Figure 3b-c.

BF bursting neurons were defined as BF single units whose average firing rates during the [0.05, 0.2]s window after stimulus onset (yellow shaded interval) increased by more than 2 spikes/s in the S^{left} sound trials compared to the corresponding window in catch trials. This contrast between sound trials and catch trials was necessary because many BF neurons changed their activity during the foreperiod while waiting for stimulus onset. In addition, BF bursting neurons should have baseline firing rates less than 10 spikes/s. The activities of BF bursting neurons in S^{left} sound trials were highly similar across sessions and across animals (Figure 3c-d).

Note that the calculation of these PSTHs to the stimulus onset event (as well as those in Figure 3b-d) did not exclude spikes that occurred after fixation port exit (as in Figures 5-7). The truncation procedure used in Figures 5-7 was needed to disambiguate BF responses to light onset from the increased BF activities after fixation port exit (i.e. evaluation responses).

Task symbols were adapted from Avila I, Lin SC (2014) Motivational Salience Signal in the Basal Forebrain Is Coupled with Faster and More Precise Decision Speed. PLOS Biology 12(3): e1001811. https://doi.org/10.1371/journal.pbio.1001811.

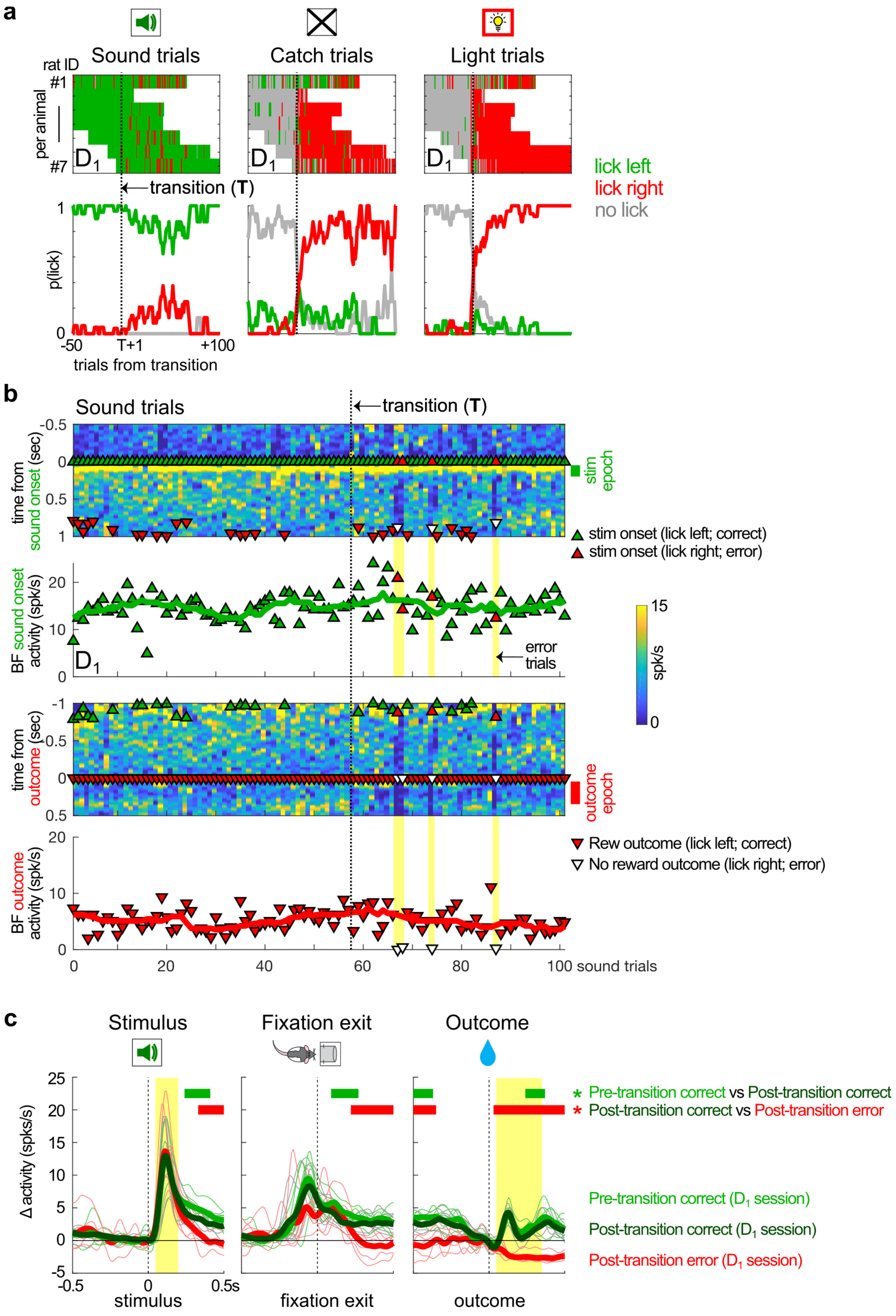

a
Sound trials
Catch trials
Light trials
rat ID
#1
#7
per animal
D1
transition (T)
lick left
lick right
no lick
1
p(lick)
0
-50
T+1
+100
trials from transition
b
Sound trials
transition (T)
time from sound onset (sec)
stim epoch
stim onset (lick left; correct)
stim onset (lick right; error)
BF sound onset activity (spk/s)
error trials
15
spk/s
0
time from outcome (sec)
outcome epoch
Rew outcome (lick left; correct)
No reward outcome (lick right; error)
BF outcome activity (spk/s)
0
20
40
60
80
100 sound trials
c
Stimulus
Fixation exit
Outcome
Δ activity (spks/s)
-0.5
0
0.5s
stimulus
fixation exit
outcome
* Pre-transition correct vs Post-transition correct
* Post-transition correct vs Post-transition error
Pre-transition correct (D1 session)
Post-transition correct (D1 session)
Post-transition error (D1 session)

Figure S3: Behavioral and BF response patterns in sound trials during the D_1 session

a, Behavioral response patterns in sound trials, catch trials and light trials during the D1 session, aligned at the behavioral transition point identified in Figure 4. All 7 animals were included in this analysis. Since the three trial types were presented at equal probabilities, the number of trials in each trial type was roughly the same. The abrupt behavioral transition also led to increased error rates (rightward licks) in sound trials, from 2.4±5.5% (mean±std, N=7 rats) in pre-transition trials to 12.0±8.4% in post-transition trials. While this increase in error rates was substantial, behavioral performance remained correct in the vast majority of post-transition sound trials in the D1 session. For comparison, the overall error rate in sound trials across sessions was 6.4±3.3% (mean±std, N=7 rats). **b**, Single trial BF responses in sound trials in the example D1 session from Figure 3e2, aligned at stimulus onset and outcome. BF activities at the two epochs were calculated at [0.05, 0.2]s after sound onset and [0.05, 0.35]s after 3rd lick. Sound trials with incorrect rightward licks were highlighted. Notice that BF responses to sound onset and reward in correct (leftward licking) trials remained fairly stable throughout the D1 session. However, BF activities in error sound trials were inhibited prior to receiving the outcome. **c**, Average BF activities aligned at the three behavioral events, plotted separately for pre-transition correct trials, post-transition correct trials, and post-transition error trials. Horizontal lines indicate significant differences in population BF activities ($p<0.01$ for 3 consecutive steps using two-sided paired t-test for each 100 ms sliding window and 10ms step). BF activity in error sound trials was significantly inhibited compared to post-transition correct trials, in the epochs prior to receiving the outcome. Task symbols in **3a** and **3c** were adapted from Avila I, Lin SC (2014) Motivational Salience Signal in the Basal Forebrain Is Coupled with Faster and More Precise Decision Speed. PLOS Biology 12(3): e1001811. https://doi.org/10.1371/journal.pbio.1001811.

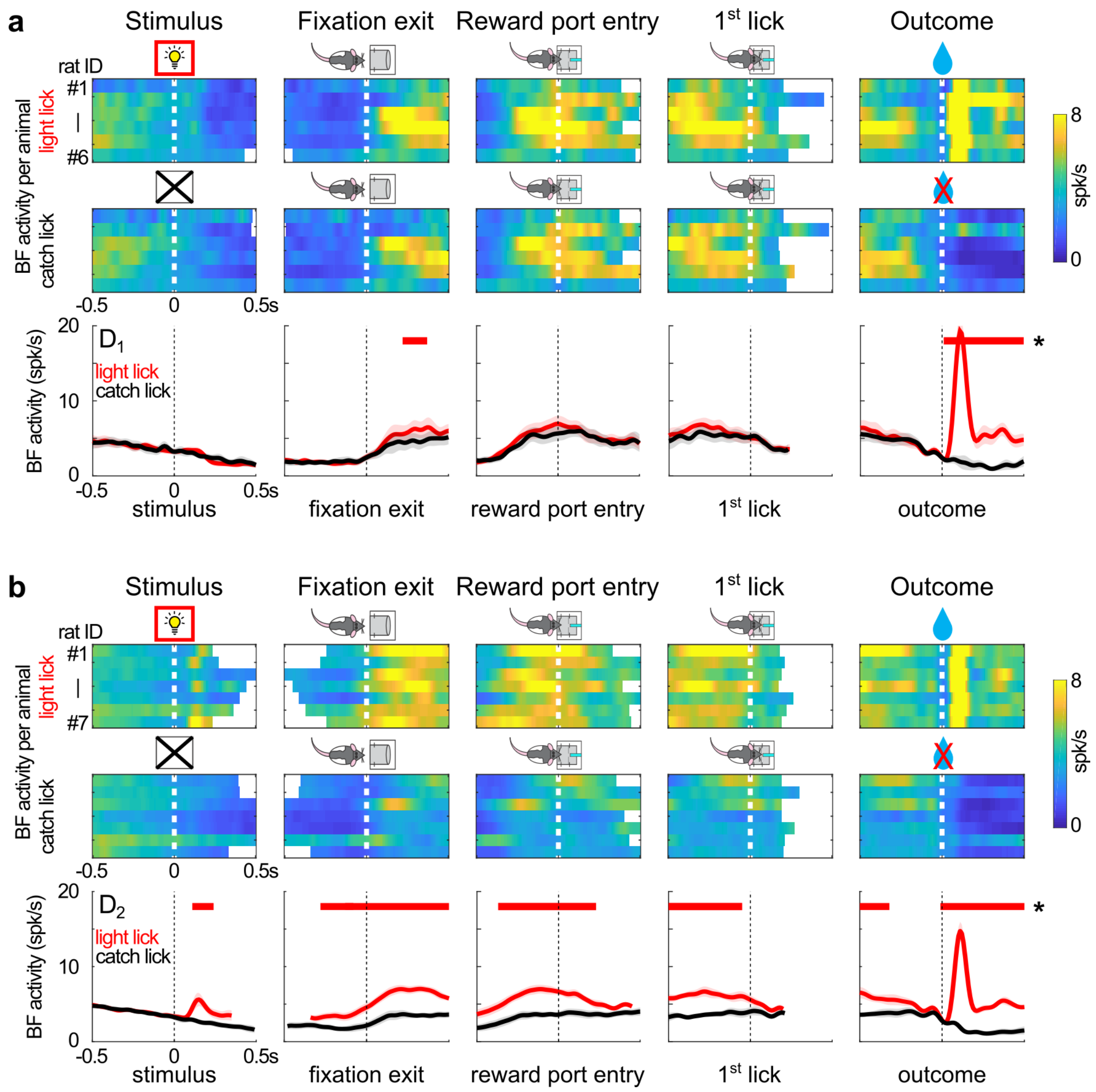

Figure S4. Variability in the timing of peak BF activities across animals and sessions

Population activities of BF bursting neurons (mean ± s.e.m.) in light lick and catch lick trials in the D_1 session (from Figure 5a) (**a**) and D_2 session (from Figure 6b) (**b**) aligned at five different events: stimulus onset, fixation exit, right reward port entry, first lick and outcome. The maximum firing rate occurred at different time points in different animals, and also varied between the two sessions. Conventions as in Figure 5a. For BF responses aligned at reward port entry and at the first lick, the calculations included only spikes that occurred after stimulus onset and before the third lick. Task symbols were adapted from Avila I, Lin SC (2014) Motivational Salience Signal in the Basal Forebrain Is Coupled with Faster and More Precise Decision Speed. PLOS Biology 12(3): e1001811. https://doi.org/10.1371/journal.pbio.1001811.

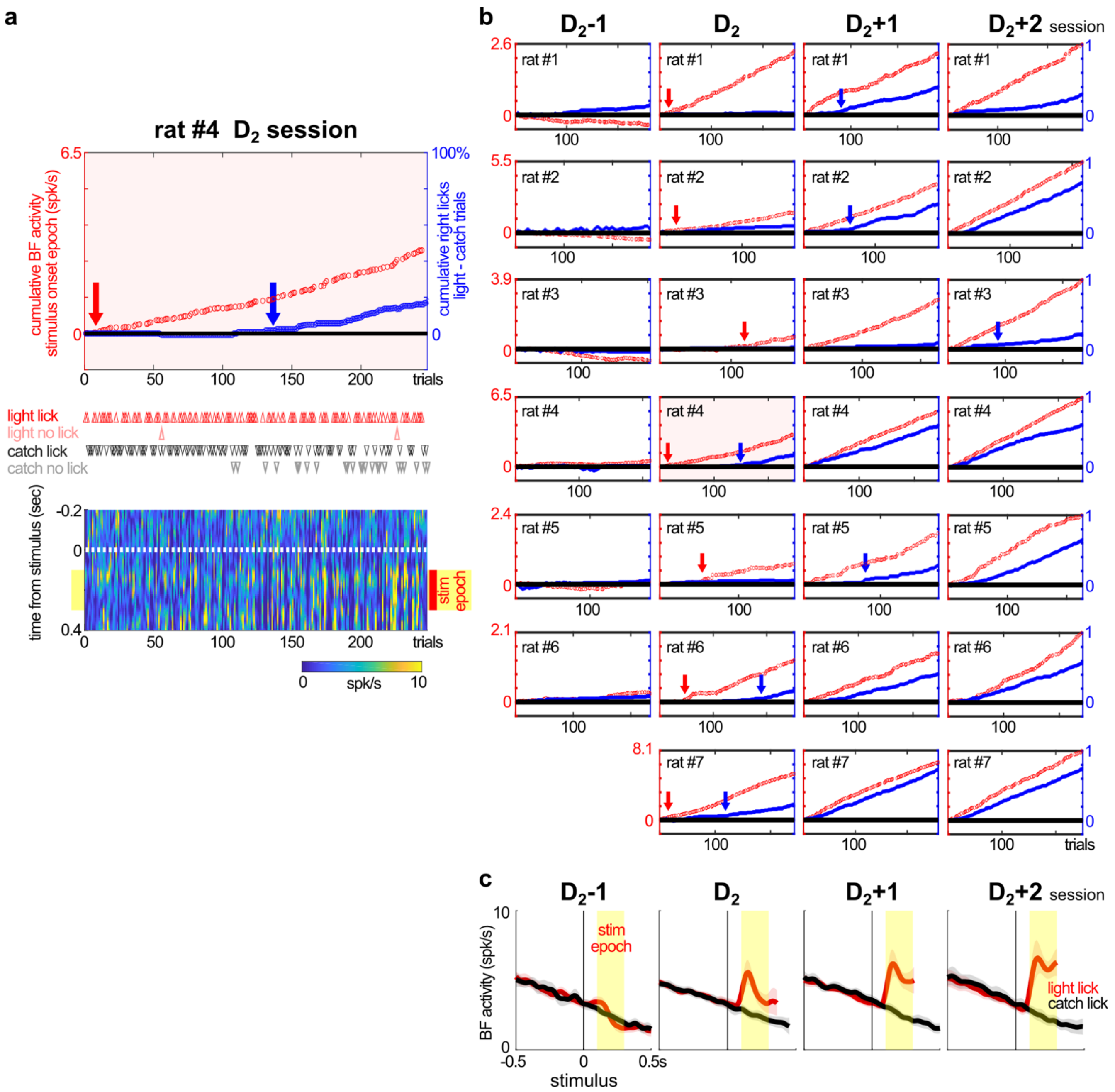

Figure S5. The emergence of BF responses to the new light preceded the elimination of catch licks

a, An example D_2 session showing how we used cumulative sums to visualize the temporal dynamics of BF responses to the new light (red) and the divergence of behavioral responses between light and catch trials (blue), plotted over the number of trials in the session (light and catch trials combined). The corresponding single-trial behavioral responses and BF activities are plotted below. BF response to the new light in each trial was defined as the difference between the average activity in the [0.1, 0.3]s window after light stimulus onset and the average activity in the corresponding window in all catch trials in the same session. The absence of BF responses to the new light would result in zero-slope cumulative sums. To determine the timing for the elimination of catch licks, we calculated the difference between the cumulative sums of

the rightward licking numbers (normalized by the number of each trial type in the session) between light and catch trials. The lack of behavioral response differences between light and catch trials would result in a zero-slope line. Thus, the timing for the emergence of BF or behavioral responses can be visualized as the divergence point of cumulative sum traces away from the horizontal line. In this example session, BF responses to the new light emerged earlier than the elimination of catch licks, indicated respectively by the red and blue arrow.

b, Each panel represents cumulative sums from one animal (each row) and one session (each column, relative to the D_2 session). To account for individual variabilities in BF response amplitudes across animals, the cumulative sums of BF responses were scaled relative to the maximum of the cumulative sum in the D_2+2 session in each animal. The increasing slopes of the cumulative sums between D_2 to D_2+2 sessions reflected the increasing response amplitudes to the new light (as shown in Figure 7a).

At the neuronal level, the lack of BF responses to the new light, which was evident in the D_2-1 sessions, resulted in zero-slope cumulative sums. On the other hand, the presence of BF responses to the new light resulted in cumulative sums with positive slopes, which first emerged in D_2 sessions (red arrows) and remained positive afterwards. At the behavioral level, the lack of behavioral response differences between light and catch trials, which was evident in the D_2-1 sessions, resulted in zero-slope cumulative sums. On the other hand, the elimination of catch licks resulted in cumulative sums with positive slopes, which emerged between D_2 to D_2+2 sessions in different animals (blue arrows) and remained positive afterwards.

Note that the timing of the emergence (red and blue arrows) were visually determined based on when the cumulative sums diverged from the horizontal line (y=0). We were not able to apply a universal criterion to rigorously define this timing because of individual differences in learning dynamics. Nevertheless, the overall pattern supports that the emergence of BF responses to the new light (all in D2 sessions) occurred earlier than the elimination of catch licks (between D2 to D2+2 sessions) in all animals.

c, The average BF activity (mean ± s.e.m.) in light lick and catch lick trials in the corresponding sessions (relative to the D_2 session). The time window ([0.1, 0.3]s) for calculating BF responses to the new light is highlighted in yellow.

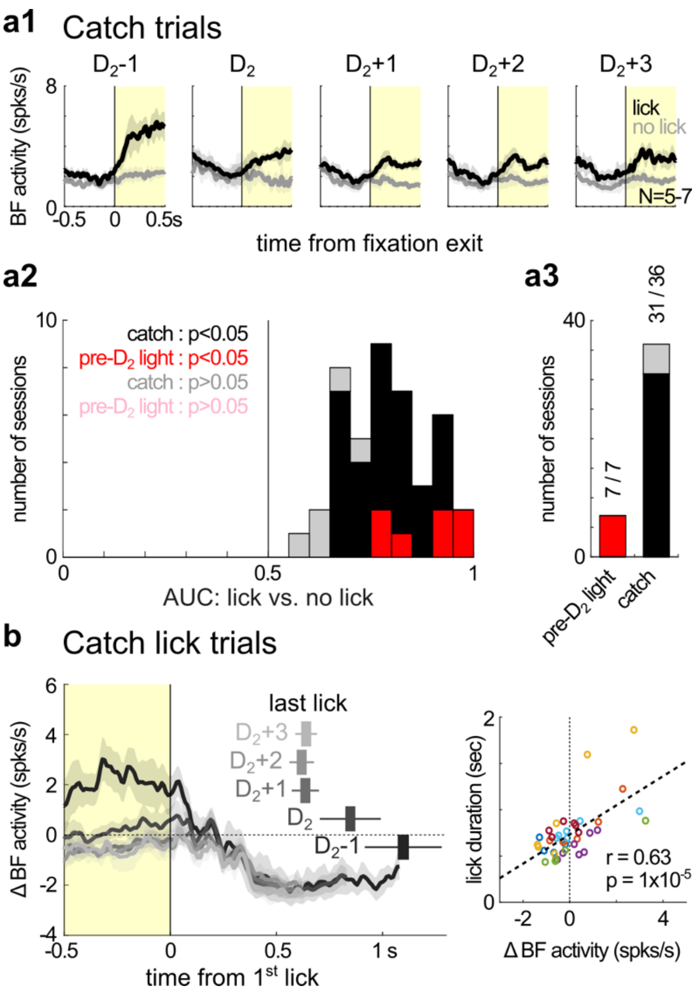

Figure S6. Increased BF activity predicted reward-seeking behaviors in the absence of the light stimulus

a1, Average responses of BF bursting neurons (mean ± s.e.m.) in catch trials aligned at fixation port exit, plotted separately for lick and no lick trials. **a2**, Distributions of AUC values from comparing BF activities in the [0, 0.5]s window after fixation port exit (yellow shaded interval in A1) between lick and no lick trials within the same trial type (catch or light trials) (two-sided AUC analysis). Within the same trial type, increased BF activities reliably predicted reward-seeking behavior toward the right reward port. Only light trials from pre-D_2 sessions were included because BF responses to the light stimulus had not developed. **a3**, Pooled histogram of all sessions in A2 to show the proportion of statistically significant sessions in light or catch trials. **b**, Average responses of BF bursting neurons (mean ± s.e.m.) in catch lick trials aligned at the first lick (left). BF activities, relative to their respective baseline firing rates, were truncated at the median lick duration of the respective sessions. The timing of the last lick (mean ± s.e.m.) of the corresponding sessions were shown above each trace. BF activities prior to the start of licking in catch lick trials (yellow shaded interval, left panel) were positively correlated (Pearson correlation) with the median lick duration in individual sessions (right). Each circle indicates one session and different colors correspond to different animals.

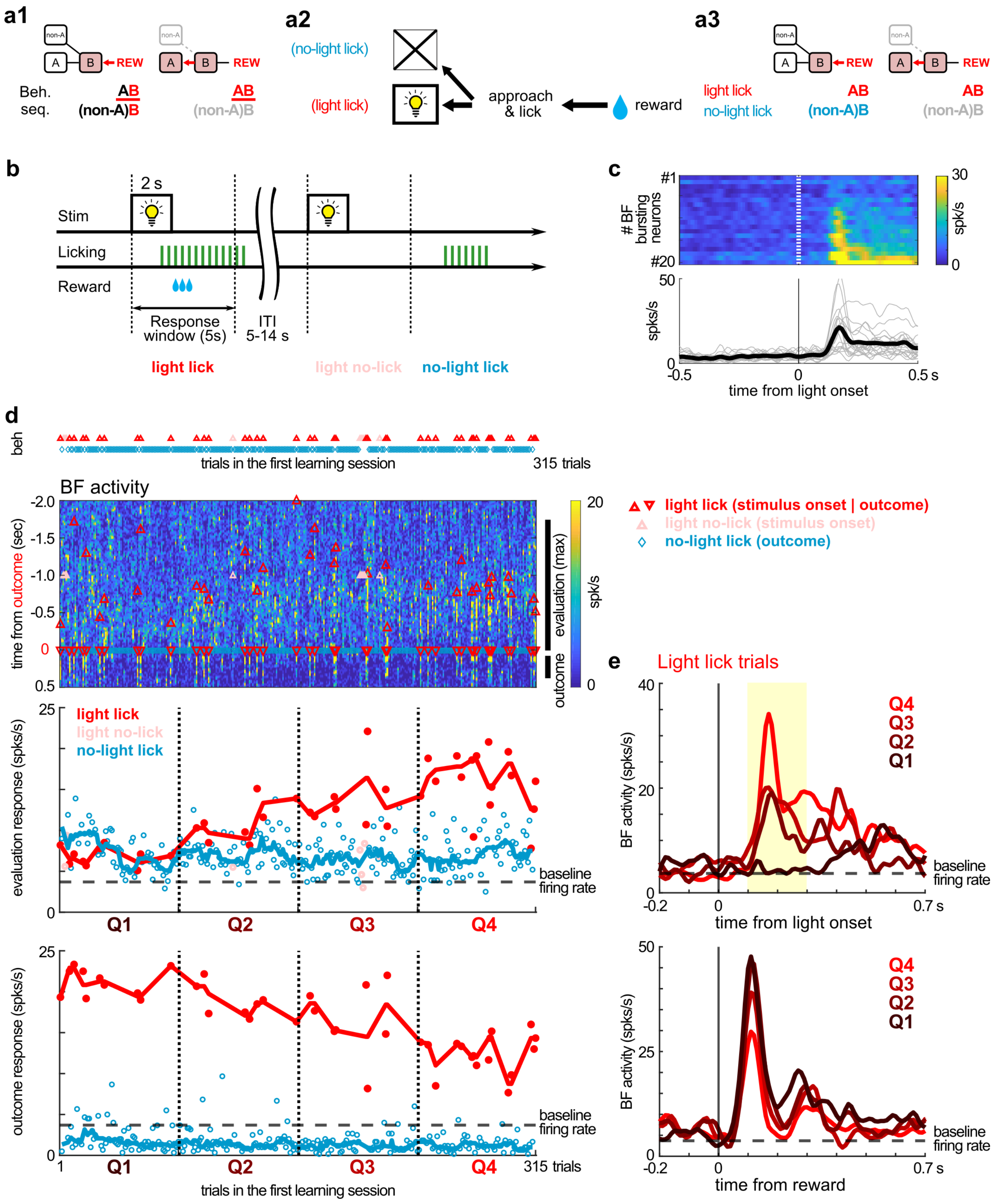

Figure S7. Stepwise learning of a two-element behavioral sequence

a, The conceptual model of how a two-element sequence is learned using the stepwise strategy. **a1**, A model depicting the two steps in learning a two-element (A-B) behavioral sequence. Conventions as in Figure 1b. **a2**, In this experiment, the A element corresponded to the light stimulus, and B the approach and licking response. Only the A-B sequence was rewarded. Task symbols were adapted from Avila I, Lin SC (2014) Motivational Salience Signal in the Basal Forebrain Is Coupled with Faster and More Precise Decision Speed. PLOS Biology 12(3): e1001811. https://doi.org/10.1371/journal.pbio.1001811. **a3**, The model predicts that, during the first step of learning, two reward-seeking behavioral sequences - light licks and no-light licks - will be observed, and both should be accompanied by similar levels of BF activities. **b**, New learning task. House light served as the reward-predicting stimulus. Licking at the reward port within a 5-sec response window led to water delivery starting at the third lick. Inter-trial interval (ITI) was randomly chosen between 5-14 sec. Licking in the absence of the light stimulus was not rewarded and reset the ITI counter. Three types of behaviors - light licks, light no-licks, no-light licks - were depicted in this schematic. No-light lick was defined as a licking cluster of at least 3 licks in length, with its first lick occuring outside the 5 sec response window of light onset, and at least 3 sec after the end of the last lick cluster. **c**, The average responses of BF bursting neurons to light onset during the first session of new learning in one example rat. 20/29 BF neurons recorded in this session were classified as BF bursting neurons based on two criteria: (1) BF activities in the [0.1, 0.3]s window after light onset increased by 2 spikes/s over baseline firing rates; (2) Baseline firing rates were within [0.1, 10] spikes/s. **d**, Behavioral and BF neuronal responses during the first learning session, plotted against trial sequence (x-axis) in this session (315 trials). **Top panel**, behavioral responses across trials. **Second panel**, population activities of BF bursting neurons across the same trial sequence (x-axis). Y-axis indicates time in each trial, with time zero aligned at the trial outcome (defined as the timing of the third lick). Light no-lick trials were aligned instead at the time of stimulus onset (pink triangles) such that the median timing of light onset in light lick and light no-lick trials were equivalent. The black lines to the right of the panel indicate the time windows for calculating evaluation and outcome responses. **Third panel**, BF evaluation responses, plotted separately for the three trial types. Evaluation response was calculated as the maximum firing rate of any 500ms window during [-1.75, 0]s before trial outcome. Circles indicate BF activities in single trials and lines indicate their respective trends (10-trial moving medians). Note that during the first quartile of trials (Q1), evaluation responses were similar between light licks and no-light licks, which became distinct in later quartiles. **Fourth panel**, BF outcome responses across trials, plotted separately for the two trial types with licking behaviors. Outcome response was calculated as the mean BF activity during [0.05, 0.35]s after the 3rd lick. Outcome responses to reward delivery became weaker over trials. **e**, Average BF responses to light onset and reward delivery in the four trial quartiles. Notice that during Q1, there was no phasic BF response to light onset. This suggests that the increased BF evaluation responses in Q1 (third panel in **d**) did not result from BF responses to the light onset, and instead reflected an internal evaluation signal that was similarly present in both light licks and no-light licks. This observation supports the stepwise learning strategy (panel **a**), with Q1 trials corresponding to the first step of learning, during which animals engaged in two types of reward-seeking behaviors - light licks and no-light licks. Light licks in Q1 trials were not driven by the light stimulus. Learning about the light stimulus occurred in later trials when BF responses to light onset emerged, which was also when BF evaluation responses began to diverge between light licks and no-light licks.
