# Supplementary material for: The behavioral signature of stepwise learning strategy in male rats and its neural correlate in the basal forebrain
Source: Nat Commun. 2023 Jul 21;14:4415. doi: 10.1038/s41467-023-40145-9 (PMC10362048; doi:10.1038/s41467-023-40145-9)
Supplement: Supplementary file 3 — Reporting Summary [file 41467_2023_40145_MOESM3_ESM.pdf]

nature portfolio

Corresponding author(s): Shih-Chieh Lin

Last updated by author(s): 2023/06/18

Reporting Summary

Nature Portfolio wishes to improve the reproducibility of the work that we publish. This form provides structure for consistency and transparency in reporting. For further information on Nature Portfolio policies, see our Editorial Policies and the Editorial Policy Checklist.

Statistics

For all statistical analyses, confirm that the following items are present in the figure legend, table legend, main text, or Methods section.

n/a	Confirmed	
☐	☒	The exact sample size (*n*) for each experimental group/condition, given as a discrete number and unit of measurement
☒	☐	A statement on whether measurements were taken from distinct samples or whether the same sample was measured repeatedly
☐	☒	The statistical test(s) used AND whether they are one- or two-sided<br>*Only common tests should be described solely by name; describe more complex techniques in the Methods section.*
☒	☐	A description of all covariates tested
☐	☒	A description of any assumptions or corrections, such as tests of normality and adjustment for multiple comparisons
☐	☒	A full description of the statistical parameters including central tendency (e.g. means) or other basic estimates (e.g. regression coefficient) AND variation (e.g. standard deviation) or associated estimates of uncertainty (e.g. confidence intervals)
☐	☒	For null hypothesis testing, the test statistic (e.g. *F*, *t*, *r*) with confidence intervals, effect sizes, degrees of freedom and *P* value noted<br>*Give P values as exact values whenever suitable.*
☒	☐	For Bayesian analysis, information on the choice of priors and Markov chain Monte Carlo settings
☒	☐	For hierarchical and complex designs, identification of the appropriate level for tests and full reporting of outcomes
☐	☒	Estimates of effect sizes (e.g. Cohen's *d*, Pearson's *r*), indicating how they were calculated

Our web collection on statistics for biologists contains articles on many of the points above.

Software and code

Policy information about availability of computer code

Data collection	Custom Med PC (Versions IV & V, Med Associates Inc) codes were used to direct animal behavior and record behavioral response timestamps. Neurophysiological signals were filtered and amplitfied using Cereplex M digital headstages and recorded using a Neural Signal Processor (Blackrock Microsystems, UT).
Data analysis	Spike waveforms were sorted offline to identify single units using the KlustaKwik sorting algorithm followed by a custom Python GUI (version 2.7) for manual curation. Data were analyzed using custom Matlab (R2018b, MATLAB The MathWorks Inc., Natick, MA) scripts. These codes are available upon request from the corresponding author.

For manuscripts utilizing custom algorithms or software that are central to the research but not yet described in published literature, software must be made available to editors and reviewers. We strongly encourage code deposition in a community repository (e.g. GitHub). See the Nature Portfolio guidelines for submitting code & software for further information.

Data

Policy information about availability of data

All manuscripts must include a data availability statement. This statement should provide the following information, where applicable:

- Accession codes, unique identifiers, or web links for publicly available datasets
- A description of any restrictions on data availability
- For clinical datasets or third party data, please ensure that the statement adheres to our policy

Source data and statistics used to make each figure are provided with this paper. The datasets analyzed in the current study are available upon reasonable request to the authors.

Human research participants

Policy information about studies involving human research participants and Sex and Gender in Research.

Reporting on sex and gender	n/a
Population characteristics	n/a
Recruitment	n/a
Ethics oversight	n/a

Note that full information on the approval of the study protocol must also be provided in the manuscript.

Field-specific reporting

Please select the one below that is the best fit for your research. If you are not sure, read the appropriate sections before making your selection.

☒ Life sciences ☐ Behavioural & social sciences ☐ Ecological, evolutionary & environmental sciences

For a reference copy of the document with all sections, see nature.com/documents/nr-reporting-summary-flat.pdf

Life sciences study design

All studies must disclose on these points even when the disclosure is negative.

Sample size	We did not perform a power analysis to determine sample size. The number of animals used were were similar to our previous studies (e.g. Mayse et al, 2015) and is sufficient to identify statistically significant correlations reported in this study.
Data exclusions	No animals were excluded from the analysis. For electrophysiology recordings, only single units with clear separation from the noise cluster and with minimal (<0.1%) spike collisions (spikes with less than 1.5 ms interspike interval) were used for further analyses. Additional cross-correlation analysis was used to remove duplicate units recorded simultaneously across multiple electrodes.
Replication	The behavioral and neurophysiology experiments were conducted on a cohort of seven rats. A separate cohort of eight rats were used for replicating the behavioral findings and for an additional recording experiment. All attempts at replication were successful.
Randomization	The first cohort of rats were initially trained with sound stimuli, and encountered a light stimulus in the new learning phase. In the second (replication) cohort, rats were initially trained with light stimuli, and encountered a sound stimulus in the new learning phase. The side (left or right) of new learning was also counter-balanced in the second cohort. Different types of behavioral trials were randomized within a session. Please see Table 1 and Figure S1 for the how animals were assigned to different groups.
Blinding	The experiment was not blinded because all animals in the same cohort received the same training protocols. All data analysis was automated.

Reporting for specific materials, systems and methods

We require information from authors about some types of materials, experimental systems and methods used in many studies. Here, indicate whether each material, system or method listed is relevant to your study. If you are not sure if a list item applies to your research, read the appropriate section before selecting a response.

Materials & experimental systems

n/a	Involved in the study	
☒	☐	Antibodies
☒	☐	Eukaryotic cell lines
☒	☐	Palaeontology and archaeology
☐	☒	Animals and other organisms
☒	☐	Clinical data
☒	☐	Dual use research of concern

Methods

n/a	Involved in the study	
☒	☐	ChIP-seq
☒	☐	Flow cytometry
☒	☐	MRI-based neuroimaging

Animals and other research organisms

Policy information about studies involving animals; ARRIVE guidelines recommended for reporting animal research, and Sex and Gender in Research

Laboratory animals	Fifteen male Long Evans rats, aged 3–6 months and weighing 300–400 grams were used for this experiment.
Wild animals	The study did not contain wild animals.
Reporting on sex	All experiments were conducted on male Long Evans rats.
Field-collected samples	This study did not involve samples collected from the field.
Ethics oversight	All experimental procedures were conducted in accordance with the National Institutes of Health (NIH) Guide for Care and Use of Laboratory Animals and approved by the National Institute on Aging Animal Care and Use Committee, and by the Institutional Animal Care and Use Committee at the National Yang Ming Chiao Tung University, Taiwan (NYCU).

Note that full information on the approval of the study protocol must also be provided in the manuscript.
